# Context-specific chromatin remodeling activity of mSWI/SNF complexes depends on the epigenetic landscape

**DOI:** 10.1038/s41392-021-00770-6

**Published:** 2021-10-06

**Authors:** Godwin Sokpor, Huu Phuc Nguyen, Tran Tuoc

**Affiliations:** Department of Human Genetics, Ruhr University of Bochum, Bochum, Germany

**Keywords:** Epigenetics, Genetics research

**Fig. 1:**
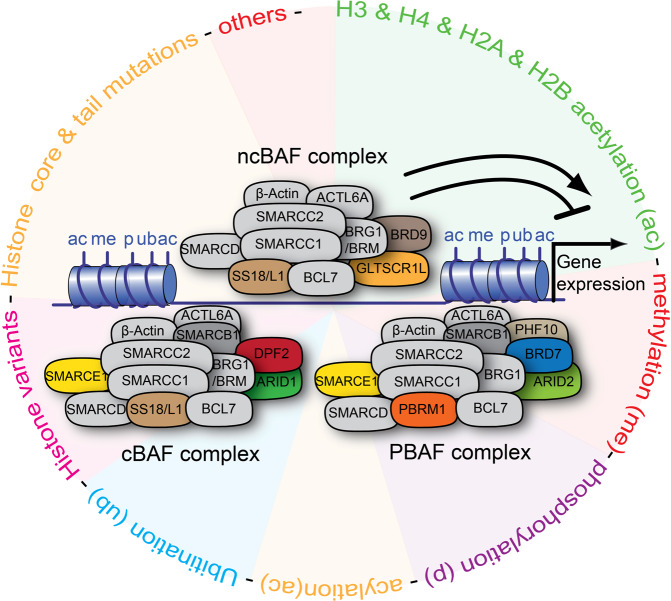
Shows a schematic illustration of the mSWI/SNF complexes and their chromatin engagement. A snapshot of the dependence of the mSWI/SNF complexes on epigenetic cues in the chromatin landscape for regulating chromatin structure; hence gene expression has been depicted. Complex-specific subunits which afford modules for differentiating the three mSWI/SNF complexes are differently color coded, whereas common subunits are given the same color codes. Chromatin accessibility is regulated by histone/nucleosome signals which drive the assembly and binding of particular mSWI/SNF complexes to exert an inhibitory or activation effect on gene expression. mSWI/SNF complexes shown: cBAF canonical BAF, ncBAF non-canonical BAF, PBAF polybromo-associated BAF. Histone marks shown: ac acetylation/acylation, me methylation, p phosphorylation, ub ubiquitylation

A recent article published in *Science* reveals how the mammalian SWI/SNF (mSWI/SNF) complexes utilize epigenetic cues in the chromatin landscape for differential chromatin remodeling.^1^ The heteromultimeric ATP-dependent SWI/SNF complexes can alter chromatin architecture leading to gene expression modulation (Fig. 1). Combinatorial and modular assembly of mSWI/SNF complex subunits^2^ partly underscore their heterogeneity and tissue-specific downstream effects. Given their implication in numerous diseases, including cancers^3^ and neurodevelopmental disorders,^4^ the mSWI/SNF or BAF complexes have attracted interest in elucidating their precise action to lend therapeutic ideas. The work of Mashtalir et al.^1^ has deepened our understanding by decoding the signals which instruct the assembly, localization, and activity of mSWI/SNF complexes. They provide experimental confirmation that the mSWI/SNF complexes depend on single and/or summative histone/nucleosome signatures and subunit modules to determine their activity (Fig. 1).

Mashtalir et al.^1^ essentially studied the remodeling activity of mSWI/SNF complexes using a repertoire of (modified) histones and wild-type/mutant histone variant nucleosomes. They purified endogenous final-form mSWI/SNF complexes (cBAF, ncBAF, and PBAF) (Fig. 1), as previously done.^2^ A battery of in vitro assays was then run to measure the binding and activity of each mSWI/SNF complex using the library of histones and nucleosome variants as substrate. A high-throughput quantitative method and advanced 3D view of nucleosome-bound mSWI/SNF complexes afforded systematic observation of mSWI/SNF functional interaction in the chromatin landscape.^1,5^ Genetic manipulation strategies, including CRISPR-Cas9-mediated gene editing, were employed in studying the contribution of specific subunits or interaction motifs/domains in the differential binding and activity of the mSWI/SNF complexes.

It is evident from the work of Mashtalir et al.^1^ that the mSWI/SNF complexes are able to contextualize epigenetic information in the chromatin landscape to regulate their remodeling function on the combinative basis of (i) the presence of specific subunits with reader modules, (ii) nucleosome engagement features, (iii) involvement of core complex modules, and (iv) the resultant complex architecture. Mashtalir et al.^1^ leveraged the characteristic presence of multiple histone reader domains and subunit dynamics in the mSWI/SNF complexes to dissect the interplay between such histone mark recognition tendencies and modalities for mSWI/SNF complex assembly.

In general, it was reported that histone posttranslational modifications or mutations which alter DNA contacts promote the activity of all mSWI/SNF complex types.^1^ Particularly, polyacetylation in the N-terminal tail of histone 3 (H3) broadly activates mSWI/SNF complex-dependent remodeling of chromatin. The acidic patch (e.g., H2A/B) also non-discriminatively potentiate the activity of mSWI/SNF complexes,^1^ and may be an amendable hotspot for differential mSWI/SNF complex-mediated chromatin remodeling.^5^ However, such cannot be said of mutations in the basic patch of the H4 tail (e.g., H4R17A, H4R19A), which were observed to confer reduced substrate preference of the mSWI/SNF complexes. A much stronger inhibitory effect on the remodeling activity of the mSWI/SNF complexes was caused by the ubiquitylation of H2A at lysine position 119. Together, these general responses to signals in the chromatin landscape give an impression of how epigenetic conditions may drive mSWI/SNF complex heterogeneity and functional plurality.

Indeed, certain chromatin features and remodeler modules underlie differential recruitment and functionality of mSWI/SNF complexes. Such signals distinctively favor the activity of one mSWI/SNF complex over the other(s). Overall, there is a moderate propensity for the binding of cBAF and PBAF to translate into activation of their remodeling functions―which correlation is reduced for ncBAF. However, cBAF remodeling activity is restricted by many modified nucleosome features that have less restrictive impact on the remodeling ability of ncBAF and PBAF.^1^ For example, methylation of H3K4 selectively blocks cBAF activity, while exerting a minimal inhibitory effect on ncBAF and PBAF. Nonetheless, in a set of experiments, the histone mark H2BK120ub (H2BK120 ubiquitylation) was seen to inhibit the binding and activity of ncBAF and PBAF, but mildly impacted that of cBAF.^1^ Interestingly, several forms of H4 acetylation, especially H4K16ac and H4K20ac, were observed to selectively promote the recruitment and activity of ncBAF at the expense of cBAF and PBAF.^1^ Of note, it has become convincing that the co-occupancy of two histone marks and (by extension) multiple histone signal localization can elicit varied signal-related consequences leading to BAF complex subtype organization, binding, and activation selectivity. This phenomenon is exemplified by the observation that the dual impact of H4 tail acetylation and H3K4me3 marks have a greater negative effect on cBAF, small inhibitory effect on PBAF, but small positive effect on ncBAF.^1^

In addition, certain reader domains and complex structural modules have been uncovered as critical for configuring mSWI/SNF complexes to discriminate signals in the chromatin landscape. Notably, the ncBAF complex-specific subunit BRD9 is identified to be responsible for its rich affinity for H4 acetylation marks compared to cBAF. Although PBAF complex, via its BRD7 subunit, also exhibits H4 acetylation binding similar to ncBAF, it cannot remodel chromatin exclusively on such basis. An argument has been made for the involvement of additional differentiating factors such as the exclusion of SMARCB1 (BAF47) subunit from the ncBAF (Fig. 1) to permit its H4 acetylation-dependent remodeling activity.^1^ On the other hand, the specificity of cBAF for certain histone mark-mediated nucleosome remodeling depends on its unique DPF2 (BAF45d) subunit, and additional adaptive modules related to its SMARCA4 (BRG1) subunit.^1^ Collectively, several chromatin modification hubs and related mechanistic nuances that orchestrate preferential remodeling activity of mSWI/SNF complexes—necessary for establishing permissive chromatin state—have been unraveled.

The current work of Mashtalir et al.^1^ has thus identified specific chromatin signatures which can individually or in a combinatorial manner selectively dictate the recruitment and activation of the various mSWI/SNF complexes. This finding partly rationalizes how chromatin remodeling could be made to occur at designated places in the genome, say, during developmental events. Future work should aim at deconstructing how the identified chromatin landscape signals influence mSWI/SNF complex function in vivo, where many more chromatin remodelers come into play. In addition, the study creates an avenue for exploring the array of mSWI/SNF complex heterogeneity within the framework of other epigenetic regulators, and in a tissue-specific manner. Investigating the effect of pathogenic mutations of various mSWI/SNF subunits on chromatin remodeling is also a promising outlook for pertinent disease modeling and therapeutic design.
